# FDG-PET/CT Incidental Detection of Cancer in Patients Investigated for Infective Endocarditis

**DOI:** 10.3389/fmed.2020.00535

**Published:** 2020-09-10

**Authors:** Frédérique Gouriet, Hervé Tissot-Dupont, Jean-Paul Casalta, Sandrine Hubert, Serge Cammilleri, Alberto Riberi, Hubert Lepidi, Gilbert Habib, Didier Raoult

**Affiliations:** ^1^Aix Marseille Univ, IRD, AP-HM, MEPHI, Marseille, France; ^2^IHU Méditerranée Infection, Marseille, France; ^3^Service de Cardiologie, Hôpital de la Timone, Marseille, France; ^4^Service de Médecine Nucléaire Hôpital de la Timone, Marseille, France; ^5^Service de Chirurgie Cardiaque, Hôpital de la Timone, Marseille, France

**Keywords:** infective endocarditis, cancer, PET/CT, incidental, diagnosis

## Abstract

**Background:** Fluorodeoxyglucose positron emission tomography/computed tomography (PET/CT) is an imaging technique largely used in the management of infective endocarditis and in the detection and staging of cancer. We evaluate our experience of incidental cancer detection by PET/CT during IE investigations and follow-up.

**Methods and Findings:** Between 2009 and 2018, our center, which includes an “endocarditis team,” managed 750 patients with IE in a prospective cohort. PET/CT became available in 2011 and was performed in 451 patients. Incidental diagnosis of cancer by PET/CT was observed in 36 patients and confirmed in 34 of them (7.5%) (colorectal *n* = 17; lung *n* = 7; lymphoma *n* = 2; melanoma *n* = 2; ovarian *n* = 2; prostate *n* = 1; bladder *n* = 1; ear, nose, and throat *n* = 1; brain *n* = 1). A significant association has been found between colorectal cancer and *Streptococcus gallolyticus* and/or *Enterococcus faecalis* [12/26 vs. 6/33 for other cancers, *p* = 0.025, odds ratio = 3.86 (1.19–12.47)]. Two patients had a negative PET/CT (a colon cancer and a bladder cancer), and two patients, with positive PET/CT, had a benign colorectal tumor. PET/CT had a sensitivity of 94–100% for the diagnosis of cancer in this patient.

**Conclusions:** Whole-body PET/CT confirmed the high incidence of cancer in patients with IE and could now be proposed in these cases.

## Introduction

Cancer in patients with infective endocarditis (IE) is not rare (1) and constitutes a special risk group with higher mortality (2). In Europe, in 2012, new cases of cancer were estimated at 3.45 million (excluding non-melanoma skin cancer), with 1.75 million deaths from cancer. Female breast, colorectal, prostate, and lung represent half of the overall burden of cancer in Europe (3). The incidence of IE is around 1.5–11.6 cases per 100,000 people (4). Furthermore, over the past 40 years, the median age of IE patients has increased. Moreover, the incidence of IE in the elderly increased 5-fold for the diagnosis of colorectal cancer compared to lung, breast, or prostate cancer (5). *Streptococcus bovis* biotype I (*Streptococcus gallolyticus*) infection is more often associated with IE (6). The link between IE and colorectal cancer was suggested in 1951 (7), and its association with *S. gallolyticus* is now recognized (6). *Enterococcus faecalis* was significantly higher in the feces of patients with colorectal cancer compared to healthy volunteers (8). Recently, a Danish nationwide study evaluated endocarditis and the risk of cancer and reported that endocarditis was an important marker of prevalent occult cancer and a predictor of a slight increase in long-term cancer risk (9).

Fluorodeoxyglucose (FDG) positron emission tomography/computed tomography (PET/CT) now plays a key role in the detection and follow-up of cancers and malignancies (10). Japan has 10 years of experience in the performance of whole-body FDG-PET in cancer screening. A large study, including 155,456 subjects involved in a PET/CT screening program, revealed the probability of cancer in 10.9% of cases. The true-positive rate was 32.3%, with a high PET/CT sensitivity for colorectal, thyroid, lung, and breast cancer and low PET/CT sensitivity for prostate and gastric cancer (11). PET/CT is non-invasive and painless and can detect cancer at potentially curable stages without targeting specific organ. However, recommendations for PET/CT cancer screening are still lacking (12, 13). The European IE management guideline suggests using PET/CT when IE diagnosis is “possible” (or “rejected”) according to the Duke criteria, but with a persisting high level of clinical suspicion (14). PET/CT is of great importance in the management of IE for the detection of metastatic infection, peripheral emboli events, and occult cancer detection (15–18).

In a 9-year prospective study, we analyzed a cohort of 750 IE patients managed in our “endocarditis team,” in which 451 PET/CT were performed. The aim of this study is to evaluate our experience of IE and cancer association, incidentally discovered by PET/CT examination performed for IE workup or follow-up.

## Materials and Methods

### Patients With IE and Cancer

From October 2009 to May 2018, we included patients with definite IE, according to the modified Duke criteria (19) and the European Society of Cardiologic criteria (14). The diagnosis was made by a multidisciplinary “endocarditis team” composed of cardiologists, cardiac surgeons, microbiologists, and pathologists.

For each case, a questionnaire was completed by the physician in charge of the patient. Data were collected upon admission or during patient hospitalization, including age, sex, signs and symptoms, duration of symptoms, history of antibiotic treatment for any current illness, previous diseases, predisposing factors for IE (prosthetic valve, systemic disease, intravenous drug abuse, dental, or surgical procedures, neoplasm), echocardiography (transthoracic and/or transesophageal), and any treatment received during hospitalization, with its outcome. The Charlson comorbidity index score was determined for each patient.

### Microbiological Diagnosis of IE

All patients had a standardized IE diagnosis (20), including blood cultures; serological testing for *Coxiella burnetii, Bartonella* spp., *Mycoplasma pneumoniae, Legionella pneumophila*, and *Aspergillus* spp.; and rheumatoid factor. Follow-up after discharge from hospital was actively carried out, either during consultations every 1, 3, or 6 months or once a year; or through transthoracic and/or transesophageal echocardiography, blood culture collection, and biological samples in our department; or by contacting patients or their doctors.

### Radiological and Nuclear Medicine Imaging

In our team, the protocol for detecting septic embolism in patients with IE includes systematic CT of the chest and abdomen and CT or magnetic resonance imaging of the brain. When it became available in 2011 and when it was possible, FDG PET/CT was performed systematically and simultaneously with these conventional diagnostic techniques when possible. After eating a high-fat, very low-carbohydrate meal, to reduce the physiological absorption of FDG into the myocardium, patients fasted for at least 12 h before PET/CT. Intravenous administration of 5 MBq/kg ^18^F-FDG was performed after the blood glucose level (<1.8 g/L) was checked. PET and whole-body CT scans were carried out consecutively using a Discovery ST PET/CT scanner (General Electric, Milwaukee, WI, USA) 1 h after ^18^F-FDG injection.

### Diagnosis of Cancer

Patients with newly discovered cancer were diagnosed simultaneously with IE (same admission) or subsequently during follow-up. An appropriate investigation was performed in case of PET/CT results suggesting cancer. All patients in the cancer group were investigated and underwent CT and biopsy, histologically diagnosed depending on the location of the tumor. Digestive investigation was systematically performed in patients with *Enterococcus* sp. or *S. gallolyticu*s IE.

### Statistical Analysis

The data were first collected from the patient's file and transcribed onto an Excel spreadsheet. The analyses were performed using R Software (version 3.2.3). Continuous variables for individuals were expressed as mean ± confidence interval and were compared using Student *t* test. Categorical variables were expressed as a percentage and were compared using Fisher *t* test. Differences were considered significant when *p* < 0.05.

## Results

### Patient's Characteristic and Incidence of Cancer in Our Cohort

**Figure 1:**
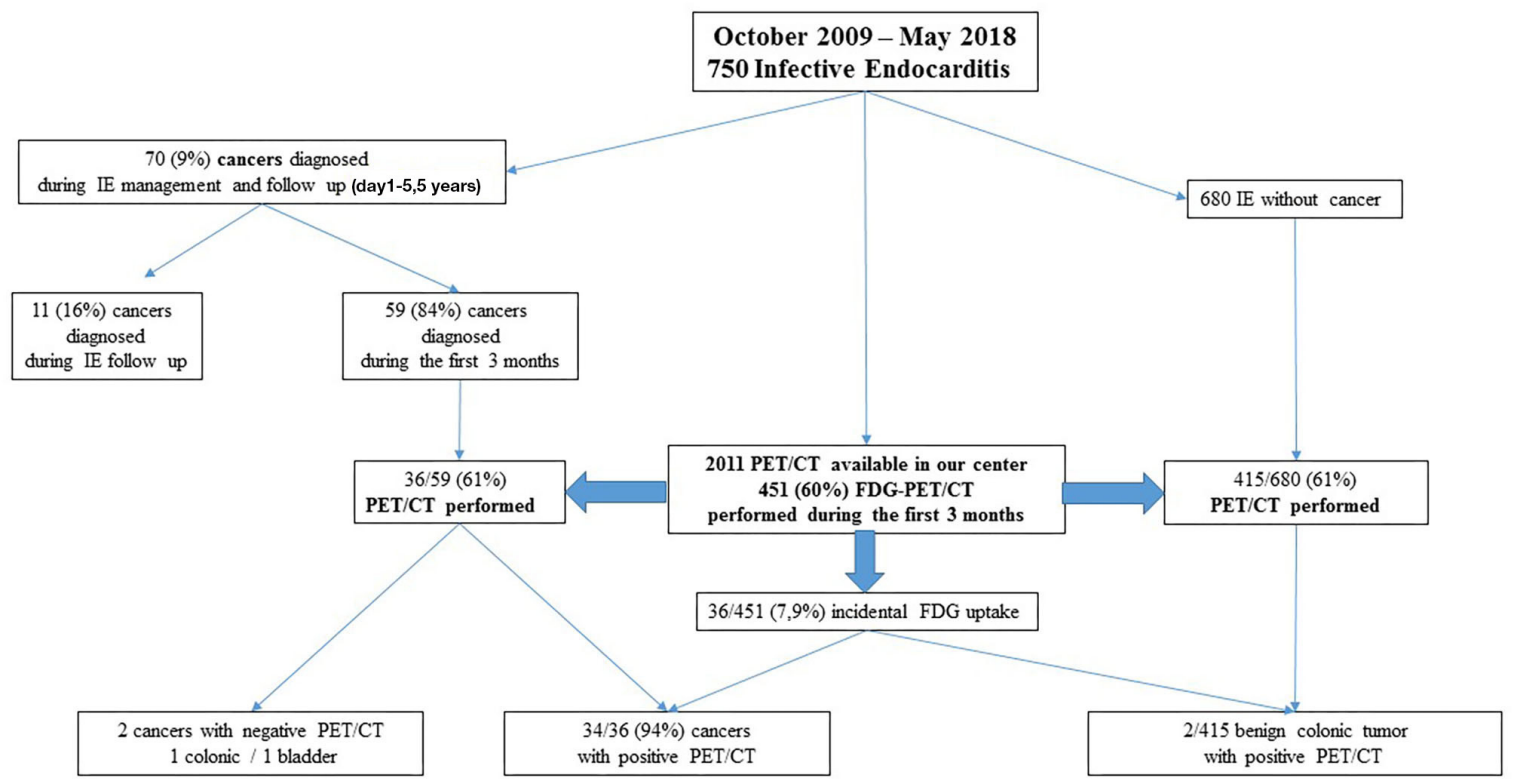
Study design. We followed 750 patients with infective endocarditis from October 2009 to May 2018. During the management at IE follow-up, incidental cancer was discovered. PET/CT was available in our center in May 2011 and was performed during the first 3 months of IE management, allowing to diagnose occult cancer.

From October 2009 to May 2018, 750 patients were diagnosed with IE and therapeutically cared for by our team. The study reported a total of 70 patients (9%) whose cancer was discovered at the time of IE management or follow-up (Figure 1). Cancer was mostly discovered at an early stage 66/70 (94%); in four cases, cancer was discovered at late stage (lung *n* = 3 and liver *n* = 1). The characteristics of IE patients with cancer and without cancer are presented in Table 1. The most common cancer reported was colorectal cancer (26/70), followed by lung cancer (4/70), prostate cancer (7/70), lymphoma (4/70), and urothelial tumors (2/70); ear, nose, and throat (ENT) cancer (2/70); and melanoma (2/70). The two groups (IE with or without cancer) were fully comparable in terms of underlying conditions. The patients tended to be older in the cancer group, the mean age at diagnosis being 68.1 ± 2.38 years old, vs. 65.2 ± 1.1 years old in the IE non-cancer group (*p* = 0.057). Although non-significant, the patients were more often male with a sex ratio (male/female) at 4.4 in the cancer group vs. 2.5 in the non-cancer group (*p* = 0.09).

**Table 1:** Patients characteristics: the characteristic of IE patients with cancer and without cancer.

**Variable**	**Newly discovered cancer**	**%**	**IE without cancer**	**%**	***p***	
**Number**	70		680			
Age (mean)	68.1 ± 2.8		65.2 ± 1.1		0.057	
**Gender**						
Male	57	81.4	486	71.5	0.09	
Sex ratio (male-to-female)	4.4		2.5			
**Underlying conditions**						
Previous IE	8	11.4	84	12.4	1	
HIV infection	2	2.9	12	1.8	0.63	
Intravenous drug abuse	5	7.1	40	5.9	0.79	
Smoking history	23	32.9	225	33.1	1	
Diabetes mellitus	8	11.4	144	21.2	0.06	
High blood pressure	30	42.9	273	40.1	0.7	
Alcohol abuse	10	14.3	71	10.4	0.42	
Atrial fibrillation	22	31.4	196	28.8	0.68	
Peripheral artery disease	8	11.4	48	7.1	0.23	
Myocardial infarction	4	5.7	75	11.0	0.22	
Stroke	7	10.0	48	7.1	0.47	
Chronic obstructive pulmonary disease	9	12.9	58	8.5	0.27	
Renal insufficiency	7	10.0	74	10.9	1	
Hemodialysis	2	2.9	20	2.9	0.63	
Previous neoplasm	17	24.3	105	15.4	0.06	
Leukemia	2	2.9	12	1.8	0.63	
Corticosteroid treatment	1	1.4	9	1.3	1	
Rheumatic disease	3	4.3	19	2.8	0.71	
Pacemaker	8	11.4	104	15.3	0.48	
Defibrillator	3	4.3	38	5.6	0.79	
Central venous catheter	4	5.7	26	3.8	0.51	
Prosthesis (bioprosthesis or mechanical)	27	38.6	252	37.1	0.9	
Congenital heart disease	3	4.3	60	8.8	0.26	
**Charlson comorbidity index**						
0	12	17.1	90	13.2	0.46	
1	4	5.7	64	9.4	0.39	
2	9	12.9	74	10.9	0.69	
3	10	14.3	138	20.3	0.27	
4+	33	47.1	314	46.2	0.9	
**Affected valve**						
Aortic	45	64.3	319	46.9	0.006	2.04 [1.22–3.40]
Mitral	27	38.6	256	37.6	0.9	
Tricuspid	3	4.3	56	8.2	0.26	
Pulmonary	1	1.4	4	0.6	0.39	
**Intracardiac device**						
Pacemaker/defibrillator	5	7.1	62	9.1	0.67	
Defibrillator	4	5.7	24	3.5	0.5	
Central catheter	1	1.4	5	0.7	1	
**PET/CT**	47	67	415	61		
Prosthesis	26/27		173/252			
**Microorganisms**						
Blood culture positive	48	68.6	600	88.2	0.00003	3.44 [1.97–5.99]
*Staphylococcus aureus*	8	11.4	164	24.1	0.016	0.41 [0.19–0.87]
Coagulase-negative *Staphylococcus*	3	4.3	80	11.8	0.069	
*Enterococcus faecalis*	7	10.0	95	14.0	0.37	
*Enterococcus faecium*	1					
*Streptococcus* spp.	26	37.1	191	28.1	0.13	
*Streptococcus gallolyticus*	13	17.1	66	9.7	0.06	
*Streptococcus mitis* group	11	15.7	117	17.2		
*Streptococcus anginosus*	1	1.4	1	0.14		
*Streptococcus agalactiae*	1	1.4	2	0.3		
*Streptococcus pneumoniae*	0		5	0.7		
*Gemella haemolysans*	0	0.0	1	0.1	1	
*Abiotrophia* sp.	2	2.9	6	0.9	0.17	
*Propionibacterium acnes*	0	0.0	5	0.7	1	
*Corynebacterium* sp.	0	0.0	3	0.4	1	
*Klebsiella* spp.	0	0.0	5	0.7	1	
*Escherichia coli*	0	0.0	10	1.5	0.61	
*Enterobacter* sp.	0	0.0	4	0.6	1	
*Serratia marcescens*	2	2.9	0	0.0	0.009	NA
*Stenotrophomonas maltophilia*	0	0.0	2	0.3	1	
*Pseudomonas aeruginosa*	0	0.0	9	1.3	0.61	
*Haemophilus influenza*e	0	0.0	4	0.6	1	
Other	0	0.0	29	4.3	0.1	
*Candida* sp.	0	0.0	4	0.6	1	
Blood culture–negative endocarditis	22	31.4	85	12.5	0.00008	3.21 [1.85–5.58]
*Bartonella henselae*	2	2.9	3	0.4	0.07	
*Bartonella quintana*	0	0.0	4	0.6	1	
*Coxiella burnetii*	0	0.0	7	1.0	1	
*Tropheryma whipplei*	0	0.0	2	0.3	1	
Nonbacterial thrombotic endocarditis	7	10.0	0	0.0	<10^−8^	NA
No etiology (previous antibiotic used)	13	18.6	69	10.1	0.04	2.02 [1.05–3.87]
**Heart surgery**	33	47.1	368	54.1	0.31	
**Total death**	21	30.0	123	18.1	0.018	1.94 [1.12–3.35]
In-hospital mortality (30 days)	5	7.1	77	11.3	0.32	
Extra-hospital mortality (44 days to 5.5 years)	16	22.9	46	6.8	0.00006	4.08 [2.17–7.69]
90 days to 1 year	7	10.0	28	4.1	0.036	2.59 [1.08–6.16]
>1 year death after	7	10.0	2	0.3	<10^−6^	37.67 [7.66–185.18]

*ENT, ear, nose, and throat; NA, not available*.

The most often affected valve in both groups was the aortic valve, especially in the IE and cancer group: 45/70 (64.3%) vs. 319/680 (50%), *p* = 0.006, odds ratio (OR) = 2.04 (1.22–3.40). Surgery was performed in 33/70 (47.1%) of the cases vs. 368/680 (54.1%), respectively. There was no difference in the in-hospital mortality rate (within 30 days), but extra-hospital mortality (44 days to 5.5 years) was four times higher in cancer patients, 16/70 (22.9%) vs. 46/680 (6.8%), *p* = 0.00006, OR = 4.08 (2.17–7.69). The mortality rate between 90 days and 1 year was 2.5 times higher in the cancer group (7/70, 10%) than in the IE group (28/680–4.1%), *p* = 0.036, OR = 2.59 (1.08–6.16). The mortality rate after 1 year was also significantly higher in the cancer group (7/63, 11%) than in the IE group (28/680–4.1%), *p* < 10^−6^, OR = 37.67 (7.66–185.18).

The etiology of IE is shown in Table 1. Positive blood culture has been significantly associated with IE without cancer group 600/680 (88.2%) vs. 48/70 (68.6%), *p* = 0.00003, OR = 3.44 (1.97–5.99). *Staphylococcus aureus* was significantly associated with IE without cancer [*p* = 0.016; OR = 2.46 (1.16–5.25)]. Although non-significant, *S. gallolyticus* was twice as common in the IE and cancer groups. A negative blood culture has been significantly associated with IE and cancer [*p* = 0.00008; OR = 3.21 (1.05–5.58)]. No etiology was found in 13/70 (18.6%) of IE with cancer vs. 69/611 (10.1%) of IE without cancer [*p* = 0.04; OR = 2.02 (1.05–3.87)], and a non-bacterial thrombotic endocarditis IE was diagnosed in 7/70 (10%) of the patients with cancer (*p* < 10^−8^). In the group of blood culture–negative endocarditis (BCNE), the positive rate of *C. burnetii* and *Bartonella* sp. IE was 8.2%, respectively (7/85 for *C. burnetii* and 7/85 4: *Bartonella quintana* and 3 *Bartonella henselae*) in the IE without cancer group and 0/22 for *C. burnetii* and 2/22 *B. henselae* in the IE and cancer groups.

### PET/CT and Cancer Detection

PET/CT was performed in 462 patients and 199 PET/CT with IE prosthetic valve, among them 26 in the IE and cancer group and 173 in the IE without cancer group.

**Figure 2:**
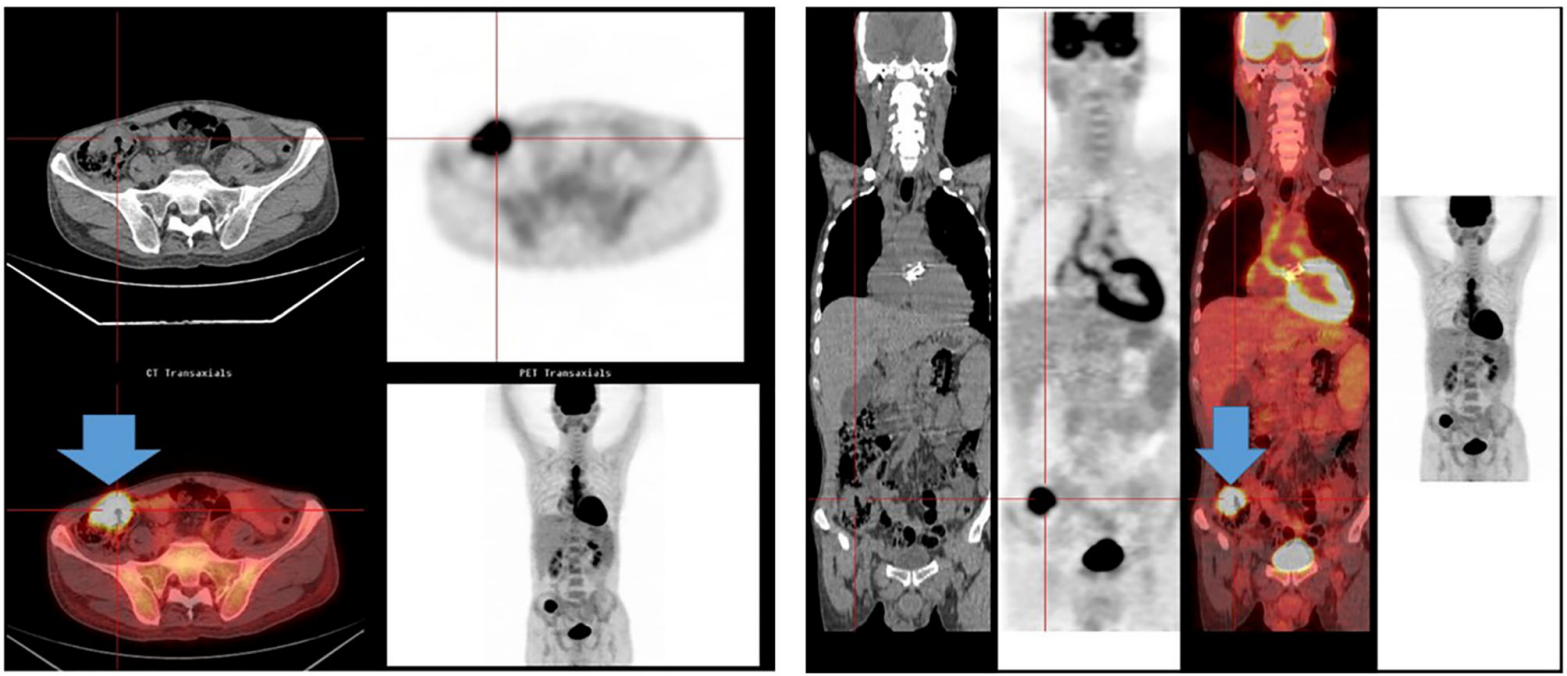
PET/CT and colorectal cancer. A 60-year-old man was admitted for *Streptococcus gallolyticus*; the PET/CT showed an intense hypermetabolic focus in the cecum, suggesting a colorectal cancer. A colorectal adenocarcinoma was diagnosed after colonoscopy.

Among the cancer and IE groups in our cohort (Figure 1), most cancer cases (59/70, 84%) (Table 2) were detected during initial hospitalization (30–90 days) of IE management (colorectal *n* = 26, lung *n* = 11, prostate *n* = 7, lymphoma *n* = 4, others *n* = 11). Eleven patients, 11/70 (16%), were diagnosed later during follow-up. PET/CT was performed in 451/750 patients (60%) during the first 3 months of follow-up. In 11 cases, diagnosis of cancer was made after the third month of follow-up between 230 and 730 days; among the 11 cases of cancer detected in IE patients, four were by a PET/CT, two by body scanner and biopsy, and two by colonoscopy. The cancer diagnosed was colorectal cancer *n* = 2, prostate *n* = 2, lung *n* = 2 bladder *n* = 2 melanoma *n* = 1, and liver *n* = 1; one patient had an ENT and lung cancer. Incidental FDG uptake suggesting cancer occurred in 36/451 cases (Table 2). Further exploration was conducted according to the FDG uptake location, such as CT or colonoscopy and specific biopsy to confirm the diagnosis. PET/CT has been effective in accidentally detecting cancer in 34 patients. PET/CT had an overall sensitivity of 94% (Figure 2). Before 2011, PET/CT was not available; in 23 patients, cancer was detected and diagnosed by body scanner and biopsy, colonoscopy, tumor markers, and mammography (colorectal *n* = 8, prostate *n* = 6, lung *n* = 4, lymphoma *n* = 2, liver *n* = 1, ENT *n* = 1, breast *n* = 1).

**Table 2:** PET/CT patients with discovered cancer diagnosed during the initial management of IE <90 days.

**Cancers**	**No. of patients**	**PET/CT done**	**Cancer detection with PET/CT**	**PET/CT Se (%)**
All cancers	59	36	34	94
Colorectal cancer	26	18	17	94
Lung cancer	11	7	7	100
Prostate cancer	7	1	1	100
Lymphoma	4	2	2	100
Melanoma	2	2	2	100
Bladder cancer	2	2	1	50
Ovarian cancer	2	2	2	100
Liver cancer	1	0	0	–
ENT cancer	2	1	1	100
Breast cancer	1	0	0	–
Central nervous system cancer	1	1	1	100

*ENT, ear, nose, and throat*.

In the IE without cancer group, PET/CT was performed in 415/680 patients (60%) (Figure 1). In 2 cases, PET/CT showed colonic focus with a final diagnosis of benign tumor by histology after colonoscopy. In the IE and cancer groups, PET/CT was performed in 36/59 patients (61%) during the first 3 months of IE follow-up. Two patients with IE and cancer had a negative PET/CT (a colonic cancer and a bladder cancer). As for colorectal cancer (confirmed by colonoscopy), PET/CT had a sensitivity of 94% and identified a colonic focus in 17/18 cases. PET/CT had a sensitivity of 100% (7/7) in lung cancer and suggested cancer in all lymphoma, melanoma, ovarian, throat, cerebral, and prostatic cancers. A bladder cancer could be identified by PET/CT in one case (1/2) because of the presence of metastasis.

In the 59 patients whose cancer was diagnosed during the hospitalization, the most frequently found microorganism was *S. gallolyticus* (11/59, 18.6%), *Streptococcus viridans* (9/56, 15.3%), *E. faecalis* (7/59, 11.9%)*, S. aureus* (6/59, 10.2%), and coagulase-negative *Staphylococcus* (3/59, 5.1%) (Table 3). Blood culture remained negative in 19/59 patients (32.2%); four cases were associated with colorectal cancer. PET/CT was performed in 2/4 and suggested colonic cancer in both cases. A significant association was found between colorectal cancer and *S. gallolyticus s*and/or *E. faecalis* IE 12/26 vs. 6/33 for other cancers [*p* = 0.025, OR = 3.86 (1.19–12.47)]. The etiology of IE in other cancers did not show any significant association.

**Table 3:** Microorganism identified in blood cultures according to the cancer diagnosed in the 59 patients diagnosed during the initial hospitalization (30–90 days) of IE management.

**Microorganisms**	**All cancer**		**Colorectal**		**Lung**		**Prostate**		**Lymphoma**	**Melanoma**	**Bladder**	**Ovarian**	**Liver**	**ENT**	**Breast**	**Brain tumor**
Total	59	**%**	26	**%**	11	**%**	7	**%**	4	2	2	2	1	2	1	1
*Staphylococcus* spp.	9	15.3	4	15.4	0	0	2	28.6	1	0	0	0	1	0	1	0
*S. aureus*	6	10.2	3	11.5	0	0	2	28.6	1	0	0	0	0	0	0	0
*Streptococcus* spp.	29	49.2	18	69.2	5	45.5	3	42.9	0	1	0	0	0	1	0	1
*S. gallolyticus*	11	18.6	8	30.8	1	9.1	1	14.3	0	1	0	0	0	0	0	0
*Enterococcus* sp.	7	11.9	4	15.4	2	18.2	1	14.3	0	0	0	0	0	0	0	0
*S. gallolyticus* and *Enterococcus* sp.	18	30.5	12	46.2	3	27.3	2	28.6	0	1	0	0	0	0	0	0
Others	2	3.4	0	0.0	1	9.1	0	0.0	0	0	0	0	0	1	0	0
BCNE	19	32.2	4	15.4	5	45.5	2	28.6	3	1	2	2	0	0	0	0

*BCNE, blood culture–negative endocarditis; ENT, ear, nose, and throat*.

## Discussion

In this study, we managed 750 patients with IE, including a significant proportion of patients with occult cancer (9%). Whole-body PET/CT was used when it became available in our hospital, and 451 patients could benefit from it (60%). In the IE and cancer groups, PET/CT had a sensitivity of 94–100% from various cancers and was most effective in detecting the most common colorectal and lung cancers. In terms of short-term prognosis, IE in cancer patients is similar to non-cancer IE; no difference in the 30-day in-hospital mortality rate was observed, although extra-hospital mortality is higher in cancer patients. A significant association was noted between BCNE and cancer. In the cancer group, a significant association was found with *S. gallolyticus* and/or *E. faecalis* and colorectal cancer.

Our data are consistent with those from the literature. Few studies are available on the prevalence of cancer in IE patients. A Spanish study reported in a 6-year study a series of 161 patients with a prevalence of 5.6% of active cancer in patients with IE (21). A nationwide Danish study including 8,444 IE patients with 997 cancers diagnosed found that patients with IE had a higher risk of cancer during the first 3 months of follow-up, particularly for liver and hematologic malignancies, compared to the general population. Between 3-month and 5-year of follow-up, the cancer incidence remained 1.5-fold higher than expected compared to the general population, with 4-fold increased for colorectal cancers (10).

A nationwide population-based cohort was conducted in Taiwanese patients with IE, showing a twice as high risk of colorectal cancer. The risks of developing overall cancer in the IE group were significantly higher than in the comparison group (22). Another large-scale prospective study in the elderly evaluated the diagnostic incidence of IE among patients with colorectal, breast, lung, and prostate cancer found that IE was more prevalent among patients with colorectal cancer (5). Whole-body PET/CT has been used to screen underlying malignancies in asymptomatic individuals. The rate of incidental cancer discovered by PET/CT in asymptomatic individuals varies from 0.74 to 3.3% (11, 22). IE appears to be associated with cancer; early detection of cancer with PET/CT could lead to more effective treatment options and could improve patient survival rates and cancer prognosis (9, 21, 22).

To the best of our knowledge, this is one of the few studies investigating the accidental discovery of cancer detected with whole-body PET/CT in the management of IE. Incidental finding was detected in 7.9% of the patients of our study (36/451), and all were further investigated: 34/36 patients (94%) with cancer and two with begin colonic tumor. In our study, the PET/CT had a sensitivity comparable to that observed in literature (11). Compared to the Japanese study, PET/CT was mostly efficient for colorectal and lung cancer (sensitivity of 89 vs. 85.9–100 vs. 86.8%). As well, colorectal and lung cancer were most frequently found (15). The role of PET/CT in IE is largely described in the literature, but we found only four studies describing occult malignancy discovered incidentally by PET/CT in patients with IE: first, a patient with possible prosthetic valve IE had a PET/CT-negative cardiac PET/CT but was diagnosed with a colon tumor with metastasis (16); second, three colorectal cancers were highlighted in a cohort of 31 patients with IE (15); third, two colonic polyp/mass was diagnosed with PET/CT in two patients, with malignant adenoma (17) confirmed with colonoscopy. Additionally, the incidental discovery of colonic focus with PET/CT should lead to a colonoscopy (23). In a recent study of 114 patients with definite IE, PET/CT identified seven (4%) unknown cancers; in two cases, this finding led to diagnostic workup and treatment modification (24).

Our findings are consistent with previous studies; the most frequently associated pathogen with IE and colorectal cancer is *S. gallolyticus* subsp. *gallolyticus* (6). In our study, in two cases of BCNE, a colorectal cancer was diagnosed by the PET/CT. In addition to colonic involvement, *S. gallolyticus* was also suggested to be related to chronic liver disease, and liver cirrhosis may progress to hepatocellular carcinoma, so this could be the relationship between endocarditis and liver cancer (25). The current European guidelines (14) recommend ruling out cancer in cases of IE caused by *S. gallolyticus*. Enterococci are an emerging cause of IE in the elderly (26), and malignancy has been found to be one of the most common comorbidities (27). Although a direct correlation between *Enterococcus* bacteremia and colorectal cancer has not yet been well-established, it will allow patients and their providers to look for each other when the other is discovered (28). In our study, 32.2% of the IE cancer group has a BCNE. In two cases, PET/CT detected a colonic focus. Cancer is a cause of non-bacterial BCNE (29). In our study, seven patients (9%) had non-bacterial thrombotic endocarditis IE with high fever, elevated inflammatory marker levels, and embolic events, suggesting IE. PET/CT can detect many types of malignant neoplasm and is widely used for the cancer check-up and follow-up (10). Moreover, the detection of cancer in IE might influence the management and the outcome of IE, especially in cardiac surgery indication, in delay of antitumor therapy.

The present study had a limitation: it was conducted in a single center. Thus, the results may not be applicable to other areas. Bias due to patient recruitment, increased surveillance, and referral policy to a tertiary cardiac center may be important. We should perform a study to evaluate the cost-effectiveness of screening cancer in IE in a further study. In France, the cost of PET/CT is around 1,000 Euros; it is lower than that in other countries. In the USA, it costs around $2,000. PET/CT cannot detect all malignancies, and the use of colonoscopy to screen colorectal cancer is still the reference methods for colorectal cancer.

Occult cancer is not uncommon in IE patients and is mainly associated with *S. gallolyticus* and BCNE. Whole-body PET/CT could be cost-effective proving the opportunity to investigate IE potential portal of entry and embolism and should be evaluated in a further study as a rapid diagnostic tool for cancer screening in the at-risk population of IE patients.

## Data Availability Statement

The datasets generated for this study are available on request to the corresponding author.

## Ethics Statement

The studies involving human participant were reviewed and approved by the Comitè de Protection des Personnes Sud-Mèditerranèe II with the number EudraCT: 2012-A01549-34.

## Author Contributions

FG managed the patients, collected validated the data, make the data analysis, and wrote the original draft preparation corrected the manuscript. HT-D validates the data, made the data analysis, and reviewed the manuscript. J-PC made the clinical investigation collected the data and managed the antibiotic treatment and validated the data. SH made the clinical investigation, validated the data, and reviewed the manuscript. SC made the PET/CT, validated the data investigation and reviewed the manuscript. AR managed the surgery, makes clinical investigation, validated the data, and reviewed the manuscript. HL made the pathologist analysis, validated the data, and reviewed the manuscript. GH made the clinical investigation validated the data and reviewed the manuscript. DR did the study design validated the data and reviewed the manuscript.

## Conflict of Interest

The authors declare that the research was conducted in the absence of any commercial or financial relationships that could be construed as a potential conflict of interest.
